# Reciprocal Packaging of the Main Structural Proteins of Type 1 Fimbriae and Flagella in the Outer Membrane Vesicles of “Wild Type” *Escherichia coli* Strains

**DOI:** 10.3389/fmicb.2021.557455

**Published:** 2021-02-12

**Authors:** Sarah A. Blackburn, Mark Shepherd, Gary K. Robinson

**Affiliations:** School of Biosciences, University of Kent, Canterbury, United Kingdom

**Keywords:** *Escherichia coli*, OMV, FimA, Flagellin, FliC

## Abstract

Fundamental aspects of outer membrane vesicle (OMV) biogenesis and the engineering of producer strains have been major research foci for many in recent years. The focus of this study was OMV production in a variety of *Escherichia coli* strains including wild type (WT) (K12 and BW25113), mutants (from the Keio collection) and proprietary [BL21 and BL21 (DE3)] strains. The present study investigated the proteome and prospective mechanism that underpinned the key finding that the dominant protein present in *E. coli* K-12 WT OMVs was fimbrial protein monomer (FimA) (a polymerizable protein which is the key structural monomer from which Type 1 fimbriae are made). However, mutations in genes involved in fimbriae biosynthesis (Δ*fimA*, *B*, *C*, and *F*) resulted in the packaging of flagella protein monomer (FliC) (the major structural protein of flagella) into OMVs instead of FimA. Other mutations (Δ*fimE, G, H, I*, and Δ*lrhA*–a transcriptional regulator of fimbriation and flagella biosynthesis) lead to the packaging of both FimA and Flagellin into the OMVs. In the majority of instances shown within this research, the production of OMVs is considered in K-12 WT strains where structural appendages including fimbriae or flagella are temporally co-expressed throughout the growth curve as shown previously in the literature. The hypothesis, proposed and supported within the present paper, is that the vesicular packaging of the major FimA is reciprocally regulated with the major FliC in *E. coli* K-12 OMVs but this is abrogated in a range of mutated, non-WT *E. coli* strains. We also demonstrate, that a protein of interest (GFP) can be targeted to OMVs in an *E. coli* K-12 strain by protein fusion with FimA and that this causes normal packaging to be disrupted. The findings and underlying implications for host interactions and use in biotechnology are discussed.

## Introduction

The role of outer membrane vesicles (OMVs) in a range of bacteria has been the subject of intense research in recent years since they were first shown in *Vibrio cholerae* by electron microscopy in the 1960’s (Chatterjee and Das, 1966; Work et al., 1966). Their diversity and ubiquity have been shown extensively and the principal foci has been on their pathogenic roles in a range of organisms with their proteomes [*Myxococcus xanthus* (Kahnt et al., 2010); *Pseudomonas aeruginosa* (Choi et al., 2011); *Campylobacter jejuni* (Jang et al., 2014)] being much more studied than their lipidomes [*Klebsiella pneumoniae* (Jasim et al., 2018); *Haemophilus influenzae and Vibrio cholerae* (Roier et al., 2016)]. Over this time, the range of cargoes that are carried by OMVs has grown, encompassing DNA (Deatherage et al., 2009), RNA (Ghosal et al., 2015), and a wide range of proteins (Horstman and Kuehn, 2002; Kaparakis-Liaskos and Ferrero, 2015).

Outer membrane vesicle formation has been speculated to play a variety of roles in intra- and inter- cellular communication as well as a specific secretion pathway (Guerrero-Mandujano et al., 2017). Strong bodies of evidence now support the hypothesis that the loading of OMVs is a regulated mechanism and does not arise due to random events nor cell death in a vast array of species (Schwechheimer et al., 2014; Schwechheimer and Kuehn, 2015). Recently, and especially since the advent of “synthetic biology,” it has also been recognized that OMVs may be beneficial for the delivery of cargo and for “synthetic” vaccines and cancer therapy using *Escherichia coli* strains (Gujrati et al., 2014; Hedari et al., 2014). OMVs are non-viable but mimic their producer cells and possess a range of beneficial features such as multiple epitopes and adjuvancy (Acevedo et al., 2014; Sanders et al., 2015).

*Escherichia coli* is the prokaryotic workhorse of microbiology and industrial biotechnology and has been sequenced and annotated across a broad range of strains to underpin resources such as the EcoCyc database^1^. Some strains of *E. coli* can also become pathogenic and cause a range of diseases such as urinary tract infections, kidney infections, cystitis, cholangitis, food poisoning, and bacteremia. Treatment for infections caused by *E. coli* is also becoming more difficult as they have developed resistance mechanisms to most first-line antibiotics (Poirel et al., 2018). Virulence factors of pathogenic *E. coli* include adhesins, flagella, fimbriae, and hemolysin. Within this study, OMVs produced by both *E. coli* K-12 and B strains are directly compared. The origin of the *E. coli* K-12 strain can be traced to a stool sample in 1922 at Stanford University (Bachmann, 1972). Although the origins of the *E. coli* B strain are unclear, it led to the widely used BL21 strains which are chemically competent and suitable for transformation (Bachmann, 1972). For the present study, it is important to note that one of the main differences between *E. coli* B strains and K-12 strains is that B strains are deficient in producing fimbriae and flagella.

While each strain might share some broad characteristics in genotype and phenotype, the variability in composition and characteristics in *E. coli* OMV formation within the literature is stark [e.g., BL21 (DE3) in Thoma et al. (2018) cf. Nissle 1917 in Hong et al. (2019)]. In many *E. coli* studies, OMV biogenesis and yield are studied post-engineering to discover what factors underpin cargo and composition for use in biotechnology and do not possess the virulence determinants (e.g., fimbriae and flagella) that are ubiquitously present in wild type (WT) strains (Lane et al., 2007; Cooper et al., 2012). We have attempted to do this herein to discover how OMV formation may be affected by the host genome and may underpin their use as a chassis for engineering cells. Importantly, we have considered the formation and composition of OMVs when structures such as fimbriae and flagella (important in motility and adhesion/invasion) are co-expressed in WT and mutant strains.

The present study focused on the effect of modifying the genome of an *E. coli* strain in an attempt to create an engineered OMV producer (i.e., that had capability to allow protein targeting). During these studies, *E. coli* K12 clearly demonstrated protein targeting [of fimbrial protein monomer (FimA) and/or flagella protein monomer (FliC)] to OMVs in particular strain backgrounds. Moreover, the mutually exclusive targeting and packaging of FimA and FliC to OMVs were shown for the first time and could be rationalized on the basis of their competing effects in host systems (Cooper et al., 2012). Using this information, the specificity of this targeting was investigated using FimA to explore the feasibility of engineering a novel OMV producer that could target cargo proteins to the arising OMVs.

## Materials and Methods

### Strains

*Escherichia coli* strains and sources were as follows:

From the Keio collection (Baba et al., 2006) strain number indicated in brackets: B Strain (#2507), MG1655 (#6300), BW2513 (#7636), Δ*fimA* (#11065), Δ*fimB* (#11063), Δ*fimC* (#11066), Δ*fimD* (#11607), Δ*fimE* (#11064), Δ*fimZ* (#11159), Δ*fimF* (#11067), Δ*fimG* (#11770), Δ*fimH* (#11068), Δ*fimI* (#11573), Δ*fliC* (#9586), Δ*lrhA* (#11785), *fliD* (#9587), *fliS* (#9588), and *flhA* (#9554).

From New England BioLabs: BL21 (DE3) (#C25271) and BL21 (#C2530H).

From Dr. Ian Blomfield, University of Kent: FimB-LacZ (#BGEC056, El-Labany et al., 2003) and Fimbriae production locked on strain (#AAEC356, McClain et al., 1993).

From Professor Sander Tans, AMOLF, Netherlands: MG1655 with FimA-GFP (Adiciptaningrum et al., 2009).

Clinical isolates, all obtained from Dr. Mark Shepherd, University of Kent. Strains 1, 5, and 6 (East Kent Hospitals University NHS Foundation (#MS207, #MS190, and #MS234 respectively). Strain 2 (#MS10, Totsika et al., 2011), Strain 3 (#MS1, Welch et al., 2002), and Strain 4 (#MS343, Klemm et al., 2006).

Further descriptions of the strain characteristics can be found in Supplementary Information 1.

### Microbial Cultivation

#### Media

All media was prepared by addition of components listed to the desired volume of distilled water which were then sterilized by autoclaving. The main media used for bacterial growth was Lysogeny broth (LB) composed of 10 g/L Bacto tryptone, 5 g/L Bacto yeast extract, and 5 g/L sodium chloride. 25 μg/ml chloramphenicol was added to LB when required for selection. Agar (Oxoid) was added (20 g/L) to media before autoclaving for the preparation of agar plates.

#### Growth Conditions

*Escherichia coli* strains were inoculated into LB and incubated at 37°C, shaking at 180 RPM for 18 h unless otherwise stated. A Shimadzu UV-1800 spectrophotometer was used to measure the optical density of cultures (at 600 nm).

### Cell Fractionation and OMV Purification

#### Standard Protocol for Purifying OMVs From Gram-Negative Bacteria

The OMV purification protocol was adapted from Nieves et al. (2010). The strain of interest was inoculated into 500 ml–1 L LB media and incubated at 37°C, 180 RPM for 18 h. The bacterial culture was pelleted by centrifugation at 12,000 RPM (14,515 × *g*) for 10 min at 4°C. The supernatant (containing OMVs) was extracted and filtered through a 0.45 μm polyethersulfone (PES) membrane filter (Nalgene Rapid-Flow) to remove any whole bacterial cells or large bacterial fragments. To ensure that all live bacterial cells had been removed, 500 μL–1 mL of filtered supernatant was spread onto LB agar plates and incubated for 24–48 h at 37°C to check for growth. OMVs were precipitated out of solution by slowly adding 1.5 M ammonium sulfate then incubated overnight at 4°C with gentle stirring. The OMVs were pelleted by centrifugation at 16,000 RPM (25,805 × *g*) for 30 min at 4°C. The resulting OMV pellets were resuspended in 10 mM *N*-2-hydroxyethylpiperazine-N-ethanesulfonic acid (HEPES)/0.85% NaCl, pH 7.4 for further analysis.

#### Standard Protocol for Purifying OMVs From Competent Cells Containing Desired Plasmid

One colony from a successful transformation was inoculated into 50 ml LB containing 25 μg/ml chloramphenicol and incubated at 37°C, 180 RPM overnight to generate a starter culture. The culture was diluted one in 100 in fresh LB with 25 μg/ml chloramphenicol (500 ml total volume). Cells were induced to express the desired plasmid by addition of 0.5 mM isopropyl β-d-1-thiogalactopyranoside (IPTG) at an OD600 of 0.25–0.3 (early stationary phase). When the induced cells had reached an OD600 of 1.0, OMVs were purified using the standard protocol for Gram-negative bacteria outlined above.

#### Outer Membrane and Periplasmic Protein Extractions

Outer membrane (OM) proteins and periplasmic proteins were isolated using Tris/sucrose/EDTA (TSE) buffer extraction (Quan et al., 2013). Isolation of periplasmic proteins, OM proteins and OMVs were performed on the same *E. coli* culture concurrently for direct comparison. In each case, the colony of interest was inoculated into 750 ml LB and grown overnight. 500 ml of this culture was used to purify OMVs and 100 ml was used for the periplasmic and OM extraction.

### OMV Characterization

#### Microscopy

##### Standard transmission electron microscopy protocol to visualize OMVs

Outer membrane vesicles resuspended in HEPES buffer were concentrated for electron microscopy (EM) by centrifugation at 13,200 RPM (14,220 × *g*) for 30 min at 4°C. The OMV pellets were resuspended in 10 μl HEPES buffer and added to a formvar/carbon-coated copper EM grid (mesh size 400) and left to settle for 10 min. OMVs were then fixed by adding 10 μl of 4% formaldehyde in PBS for 10 min. The grids were subject to 4 × 1 min water washes then negatively stained using 2% uranyl acetate in water. Grids were air dried and loaded on to the Jeol transmission electron microscope (model JEM 1230). Photos taken using a Gatan multiscan digital camera and operated at an accelerating voltage of 80 kV.

##### Embedding bacterial cells and omvs in resin for immunogold labeling and TEM analysis

Methods used for embedding bacterial cells and OMVs in resin for immunogold labeling were developed based on a protocol in the literature (Lee et al., 2016). See Supplementary Information 2 for details on embedding, sectioning, immunogold labeling and visualization of embedded samples.

#### Protein Manipulation Techniques

##### Bradford assay

The concentration of protein in cells, outer membrane fractions, periplasmic fractions and OMV samples were determined using a Bradford assay. Bradford reagent (Bio-Rad catalog # 5000006) was used and the assay was performed as per the manufacturer’s instructions.

##### TCA precipitation of OMVs

Purified OMVs [resuspended in HEPES (HEPES is 10 mM + 0.85% NaCl adjusted to pH7.4 and filter sterilized)] were thoroughly mixed with cold 100% trichloroacetic acid (TCA) stock solution (Sigma-Aldrich catalog #T4885) to make a final concentration of 20% TCA. Samples were incubated on ice for 30 min then centrifuged at 13,200 RPM (14,220 × *g*) for 30 min at 4°C. The supernatant was removed and the pellet was resuspended in 0.5 ml ice-cold acetone. The samples were centrifuged at 13,200 RPM (14,220 × *g*) for 15 min at 4°C. The supernatant was removed and each pellet was resuspended in HEPES and 4x RSB (Reducing Sample Buffer, Invitrogen catalog #NP0008) in a 3:1 ratio.

##### SDS-PAGE

Samples were standardized to the same protein concentration then subject to TCA precipitation to concentrate. Each sample was then mixed with the appropriate volume of 4x RSB and heated to 95°C for 5 min. SDS-PAGE (sodium dodecyl sulfate–polyacrylamide gel electrophoresis) gels were run using the Invitrogen Novex Xcell II Mini-Cell system for Electrophoresis with NuPAGE pre-cast 10 well 4–12% Bis-Tris gels. 20 μl of each sample was loaded into each well. 0.2 μl markers (Bio-Rad, catalog #1610374) were used each time to estimate protein size when visualized using silver staining and 5 μl for Western blotting. Gels were run at 165 V for 48 min in MES [2-(N-morpholino) ethanesulfonic acid] running buffer or 55 min in MOPS [3-(N-morpholino) propanesulfonic acid] running buffer.

##### Detection of proteins *via* silver staining

SDS-PAGE gels were developed using the Pierce Silver Stain kit (Thermo-Fisher catalog #24612) as described in the manufacturer’s protocol.

##### Western blot protocol

Transfer of proteins from an SDS-PAGE gel to a PVDF membrane was performed using the Bio-Rad electro transfer cell equipment (catalog #1703930) following the manufacturer’s instructions. All steps below were carried out on an orbital shaker (Stuart Scientific). After transfer, the membrane was blocked in 5% (w/v) milk powder in 10 mM Tris, 137 mM NaCl, 0.1% Tween 20 at pH 7.4 (TBST) for 30 min then incubated overnight at 4°C with primary antibody diluted in 5% milk in TBST (see Supplementary Information 3 for details of dilutions for each primary antibody). Membranes were then subject to 4 × 5 min washes in 1X TBST then incubated for 1 h with secondary antibody (diluted 1:5,000 in 5% milk in TBST). Membranes were then subject to 4 × 5 min washes in 1X TBST. Bands were developed in the dark using BCIP/NBT substrate (Sigma-Aldrich) for 1–10 min.

##### Imaging of SDS-PAGE gels and western blots

Gels and blots were imaged using Syngene G:BOX and associated software.

##### Mass spectrometry (matrix-assisted laser desorption/ionization, MALDI).

SDS-PAGE gels containing the bands of interest were subject to 2 × 10 min washes with ultrapure water. Bands of interest were then carefully excised from the SDS-PAGE with a clean washed scalpel and cut further into 1 mm × 1 mm squares. A protocol for in-gel digestion was carried out as described in Shevchenko et al. (1996). Proteins were identified using Bruker ultrafleXtreme MALDI-TOF/TOF mass spectrometer and associated software. See Supplementary Information 4 for further information.

##### Proteinase K test

The protocol for the Proteinase K test was adapted from McCaig et al. (2013). Purified OMV samples were treated with a working concentration of 10 μg/ml Proteinase K (resuspended in 10 mM HEPES/0.85% NaCl/20 mM CaCl_2_ buffer, pH 7.4) and/or varying concentrations of SDS (in sterile ultrapure water). The OMVs (resuspended in HEPES buffer) were incubated in the presence and absence of Proteinase K and SDS for 30 min at 37°C. Phenylmethylsulfonyl fluoride (Sigma-Aldrich) was added to every sample (at a working concentration of 0.5 mM) to inhibit Proteinase K and incubated for 30 min at 37°C. Samples were TCA precipitated to concentrate for loading on to an SDS-PAGE gel.

### Cloning

#### Plasmid Construction

A plasmid (pSB001) was constructed to produce a FimA-mNeon Green protein fusion with a N-terminal hexa-histidine tag. Primers used were from Integrated DNA Technologies (IDT). Further plasmid and primer information can be found in Supplementary Information 5.

#### Preparation of Competent Cells

*Escherichia coli* parental BW25113 (CGSC #7636) and *E. coli* Δ*fimA* (CGSC #11065) were made chemically competent using standard protocols (Ausubel et al., 1994).

#### DNA Manipulation

The cloning techniques used in this study were carried out using standard protocols (Ausubel et al., 1994). Plasmid DNA was isolated using the Plasmid Miniprep kit (Qiagen) and *Asc*I and *Nde*I restriction enzymes were purchased from New England BioLabs. PCR reactions were performed in Veriti 96 Well Thermocycler (Applied Biosystems). Agarose gels were prepared using the multiSUB Midi electrophoresis unit, 10 cm × 10 cm UV Tray, 2 × 16 sample combs, loading guides, and dams. 1 or 2% agarose gels were run at 150 V for 25 min on the Fisherbrand multiSUB Midi Horizontal Gel System then stained for 30 min in 0.5 μg/ml ethidium bromide in ultrapure water. Bands were visualized using G:Box machine by SynGene and associated software. Lastly, samples were sent to Genewiz for Sanger Sequencing^2^.

## Results

### Characterizing the Proteome of *Escherichia coli* K12 and *Escherichia coli* B Strains

**FIGURE 1:**
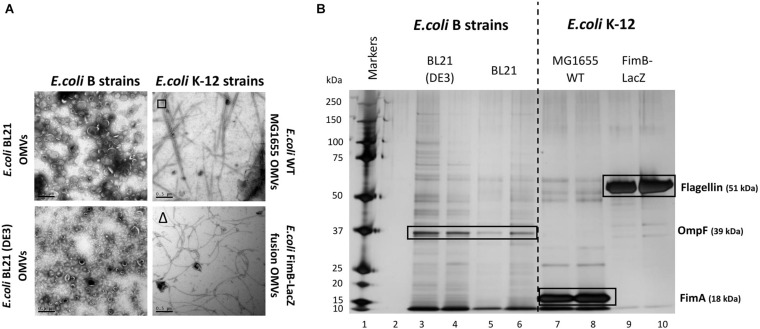
Comparison of the OMV yield **(A)** and proteome **(B)** from *E. coli* K-12 wild type MG1655 strain, FimB-LacZ fusion strain (where fimbriae production is locked off) vs. B [BL21 and BL21 (DE3)] strains. OMVs were purified concurrently for a direct comparison. Samples were from the same harvest points and with equivalent gel loading concentrations. It is noted that OMVs from *E. coli* K-12 strains were co-purified with either flagella (Δ) or fimbriae (□).

There have been many studies of OMV formation in a range of bacterial species and strains. Herein we investigated how different *E. coli* strains, the major chassis for many biotechnological processes, vary in OMV yield and composition. All *E. coli* strains were grown at 37°C, 180 RPM for 18 h to reach late stationary phase. OMVs were purified concurrently from two recombinant *E. coli* B strains: BL21 and BL21 (DE3) and two *E. coli* K-12 strains: *E. coli* WT MG1655 and *E. coli* FimB-LacZ fusion strain (where fimbriae production is locked off). As outlined in the Introduction, these strains differ in their production of fimbriae and flagella and the methods used to distinguish between the two are outlined in Supplementary Information 6. They are absent in BL21 strains but present in the K12 strain MG1655 (which expresses Type 1 fimbriae) and the FimB-LacZ protein fusion strain which possesses flagella (as fimbriae production is locked off) (Figure 1A).

At present, the variability in the proteins associated with *E. coli* OMVs is not well documented, especially when comparing WT strains with proprietary strains. As shown in Figure 1, there is a significant difference in both the OMV yield (Figure 1A) and the OMV protein profile (Figure 1B) of the WT K12 strains when compared with the *E. coli* B strains [BL21 and BL21 (DE3)]. Both *E. coli* BL21 and BL21 (DE3) have a diverse array of proteins when compared with K12 strains that have clearly demonstrable enrichment of very few proteins (Figure 1B). These proteins were analyzed by mass spectrometry and the results are shown in Supplementary Information 7 and Figure 1. As highlighted, it is shown that the dominant and identifiable proteins were monomeric FimA (the main structural protein of Type 1 fimbriae) in *E. coli* MG1655 OMVs and Flagellin/FliC (main structural protein of flagella) in *E. coli* FimB-LacZ fusion protein OMVs. Interestingly, the packaging of FimA and FliC within *E. coli* K-12 OMVs appeared to be mutually exclusive.

### FimA (Type 1 Fimbriae Major Subunit) and FliC (Major Flagella Filament Structural Protein) Are Reciprocally Packaged Into Wild Type *Escherichia coli* OMVs

**FIGURE 2:**
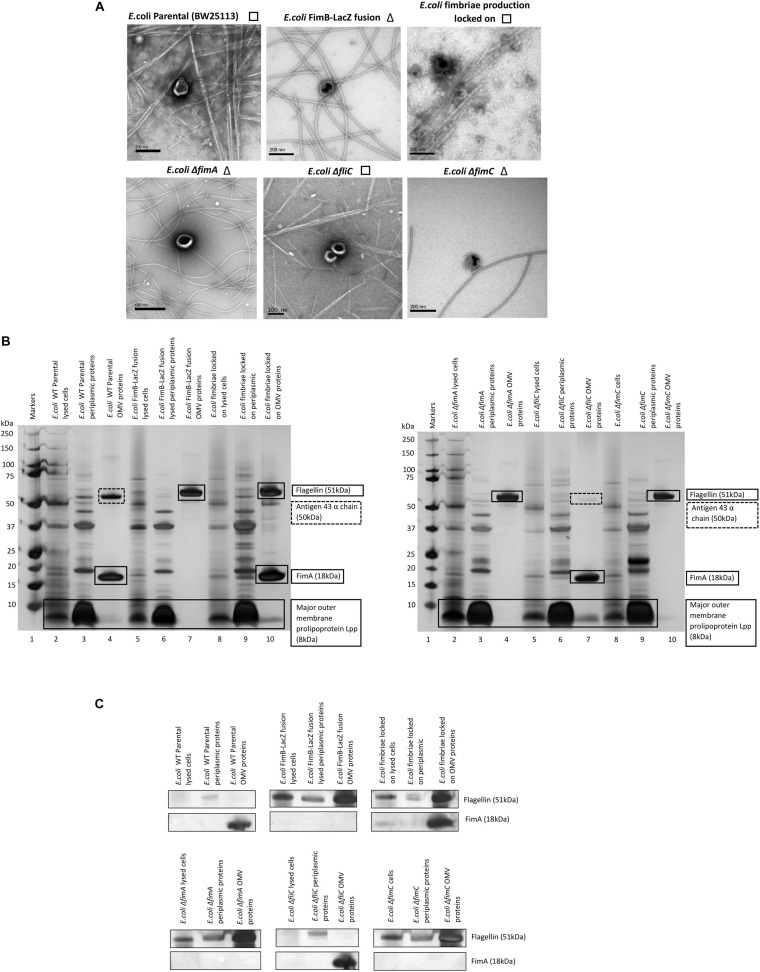
Enrichment and location of preferentially packaged proteins in the OMVs of *E. coli.* The co-purification of either fimbriae (□) or flagella (Δ) but not both is highlighted next to TEM images of OMV preparations derived from K12 strains **(A)**. FimA and Flagellin are enriched in *E. coli* K-12 OMVs compared to levels in the periplasm and whole cell **(B,C)**.

Using different strains, we investigated further whether FimA or FliC is present in *E. coli* K-12 whole cells, periplasmic fractions and OMVs. When comparing a K-12 WT strain (BW25133) with a *fimA* or *fliC* mutant, it can be seen that either FimA or FliC is enriched in the OMV samples (Figures 2A,B). Additionally, if the abundance of each of the monomers (FimA or FliC) is compared with whole cells and periplasmic fractions, it is evident that FimA (in BW25113 and Δ*fliC*) and FliC (in Δ*fimA*) are enriched in the OMV samples (Figures 2B,C).

It can also be concluded from these results that FimA is the key protein in the *E. coli* K-12 (BW25113) OMV proteome. However, the main cargo found in the OMV switches from FimA to FliC when there is a single gene deletion (Δ*fimA*) or the regulation of Type 1 fimbriae is disrupted (FimB-LacZ strain) i.e., there is a switch from the most abundant structural protein in fimbriae (FimA) to the most abundant structural component of flagella (FliC). See Supplementary Information 7 for mass spectrometry details. A Proteinase K test was also performed to distinguish which proteins are outside the OMVs and which are protected within the lumen. These experiments clearly showed that flagellin (FliC), but not FimA, was accessible and hydrolyzed by Proteinase K only when SDS was present to disrupt the integrity of the OMVs (see Supplementary Information 8).

### Abrogation of Fimbrial Assembly Alters OMV Cargo

**FIGURE 3:**
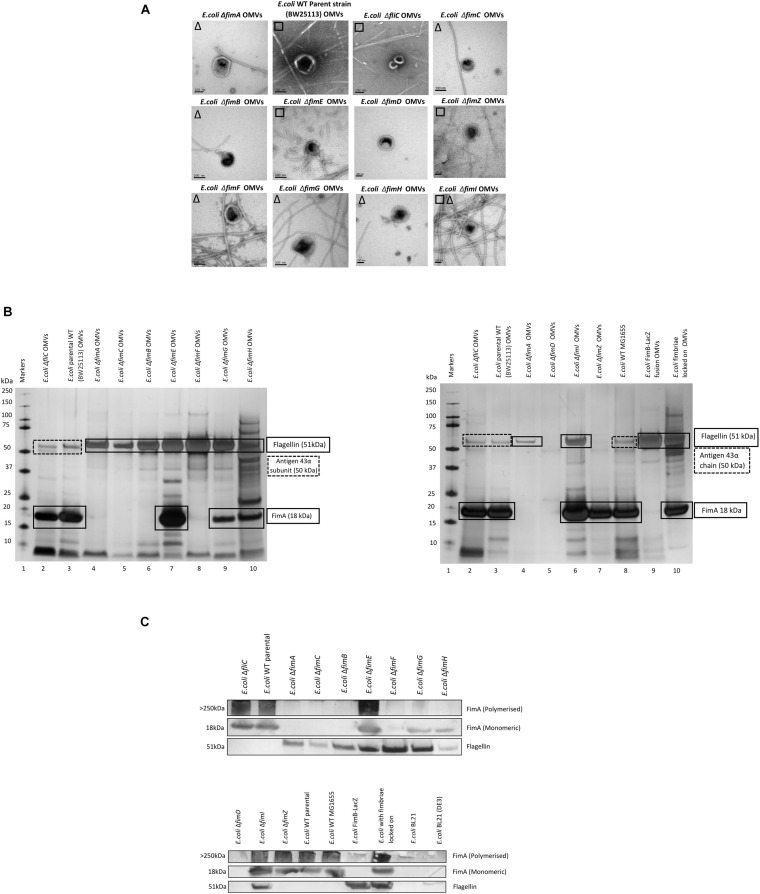
The effect of mutations in various genes of the *fim* operon using WT (*E. coli* BW25113) and mutants from the Keio collection. **(A)** OMV co-purification with either fimbriae (□) or flagella (Δ); **(B)** Proteome of the OMVs labeled with proteins identified by mass spectrometry **(C)** Western blot using anti-FimA (monomer and polymerized) and anti-Flagellin antibodies.

The biosynthesis of fimbriae in *E. coli* is controlled by the *fim* operon which co-ordinately regulates 8 genes (*fimA*-H) *via* the invertible 314 bp *fim* switch comprising the recombinases FimB and FimE (Gally et al., 1994). Using the Keio collection (Baba et al., 2006), we investigated how different deletion mutants would alter both the formation of the OMVs and the proteomes therein. While it may have been expected that the biosynthesis of fimbriae and the loading of “monomeric” FimA into OMVs is linked, it is not a clear-cut story. Using the bank of Keio mutants in conjunction with the WT strain (BW25113), it can be seen that all form OMVs but the presence or absence of either fimbriae or flagella varied (Figure 3A). It may have been expected that any mutant that affects the formation of flagella (such as Δ*fliC*) leads to fimbriae formation and any mutant that effects fimbriae formation (Δ*fim*A, B, C, D, E, F, G, H, I, and Z) leads to flagella formation but this was not shown with Δ*fimE* and Δ*fimZ* producing fimbriae and Δ*fimI* appearing to produce both fimbriae and flagella.

The proteomes of the OMVs from each mutant strain were examined and this is shown in Figures 3B,C, Supplementary Information 7, and Figure 3. Two antibodies were used to distinguish between monomeric FimA (which has the potential to be OMV-associated) and polymerized FimA (which forms the main structure of Type 1 fimbriae). Monomeric FimA was detected at 18 kDa while remaining undenatured polymeric FimA was detected in the wells of the SDS-PAGE gel. This is because the structure of type 1/polymerized FimA fimbriae is too large to migrate through the gel and therefore this is the area examined after Western blotting (Figure 3C). In the WT strains, the arising OMVs contained monomeric FimA as the dominant protein (and no FliC present). Mutants in *fimA*, *fimB*, *fimC* (periplasmic chaperone for fimbrial proteins) and *fimF* (fimbrial tip protein) altered the OMV proteome which was shown to contain FliC as the major protein (with no FimA present). Interestingly, mutations in *fimE* (fimbrial production locked off switch), *fimG* (fimbrial minor subunit), *fimH* (adhesin on fimbrial tip), and *fimI* (*fimA* homolog with unknown function) disrupted the exclusivity leading to both FimA and FliC being packaged into the OMVs.

Polymerized FimA (within Type 1 fimbriae) was detected in the following samples: WT (BW25113), Δ*fliC*,Δ*fimE*,Δ*fimI*,Δ*fimZ* [activator of the promoter to express the *fim* structural genes in *Salmonella* (Saini et al., 2009)], MG1655, and fimbriae locked on strains. Interestingly, Figure 3C indicated that Δ*fimG* and Δ*fimH* OMV samples contained no polymeric FimA but did contain FimA monomer. These results suggested that the FimA monomers are packaged independently of fimbrial formation and/or packaging of OMVs is coordinately regulated and abrogation of that control leads to mispackaging. Δ*fimE* and Δ*fimI* are key to this demonstrating that altered regulation leads to both FimA (monomers) and Flagellin (monomers) being packaged in the OMVs. See Supplementary Informations 9, 10 for summary tables of these findings and Supplementary Information 11 for further discussion of each finding.

### The Effect of Regulatory Mutants on the Composition of OMVs and Co-purified Appendages in *Escherichia coli* WT (BW 25113)

**FIGURE 4:**
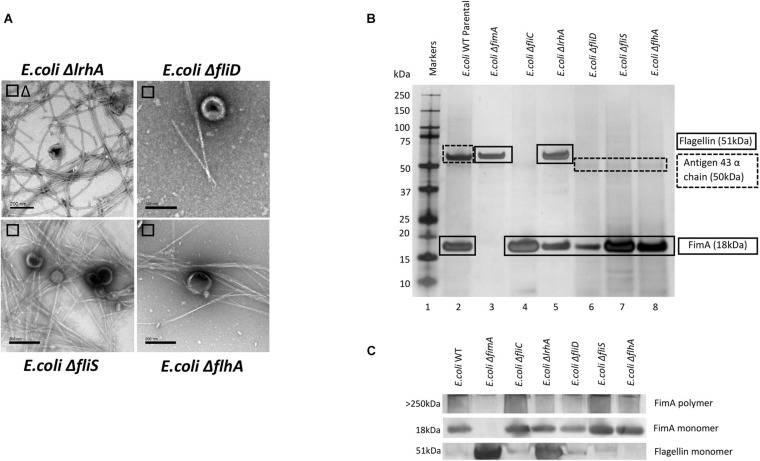
The effect of regulatory mutants on the composition and co-purified appendages in *E. coli* WT (BW25113). **(A)** OMV co- purification with either fimbriae (□) or flagella (Δ). **(B)** Proteome of the OMVs and proteins identified by mass spectrometry **(C)** Western blot using anti-FimA (monomer and polymerized) and anti-Flagellin antibodies.

It might be expected that other control systems, known to regulate the synthesis of either fimbriae or flagella, also modulate the packaging of cargo into OMVs. If disrupted, this may cause the dysregulation of the packaging of FimA and FliC into OMVs. As can be seen in Figures 4B,C, only Δ*lrhA* (a deletion in a key transcriptional regulator of fimbriation and flagella biosynthesis *via* the master regulator FlhDC, Lehnen et al., 2002; Blumer et al., 2005) caused dysregulation where both FimA and FliC were packaged into OMVs (see Supplementary Information 7 and Figure 4 for MS identification). It can also be seen that Δ*lrhA* caused production of both fimbriae and flagella on the OMV-producing cell. Lastly, OMVs were also purified from *E. coli* strains containing knockouts of various proteins associated with flagella biosynthesis: Δ*fliD*,Δ*fliS*, and Δ*flhA.* Absence of these genes had no effect on the packaging of FimA in the OMVs produced (Figures 4B,C).

### Investigating Mutual Exclusion in *Escherichia coli* Clinical Isolates

**FIGURE 5:**
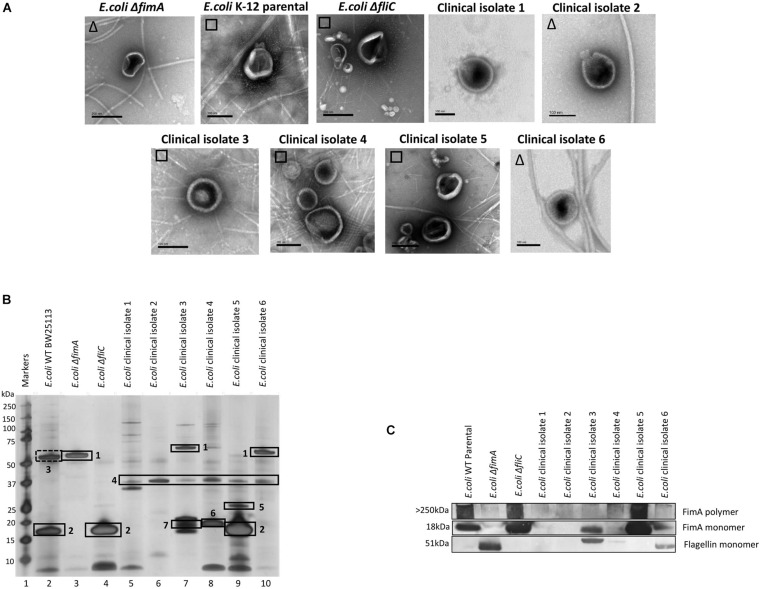
The ubiquity of mutual exclusivity using *E. coli* WT, deletion mutants (Δ*fimA* and Δ*fliC*) and a range of *E. coli* clinical isolates (described in section “Materials and Methods”). **(A)** OMV co-purification with either fimbriae (□) or flagella (Δ). **(B)** Proteome of the OMVs and **(C)** Western blot using anti FimA (monomer and polymerized) and anti-Flagellin antibodies. Proteins identified in panel **(B)**: 1, Flagellin; 2, FimA; 3, Antigen 43α chain; 4, OmpA; 5, FimH; 6, KS71A fimbrillin; 7, F7-2 fimbrial protein precursor.

Having demonstrated that FimA and FliC appear to be packaged in a mutually exclusive way, it was interesting to speculate whether this was also true of clinical isolates. In a small study, six clinical isolates were examined using EM, SDS-PAGE, Western blotting and mass spectrometry to ascertain whether FimA and/or FliC monomers were packaged in OMVs (Figures 5A–C and see Supplementary Information 7; Figure 5 for MS identifications). Clinical isolate 5 OMVs contained FimA monomer and no FliC and Clinical isolate 6 OMVs contained FliC but no FimA monomer, which fits the mutual exclusivity hypothesis. However, Clinical isolate 3 contained both FimA and FliC, which means that the mutual exclusivity theory is not a clear picture. Further discussion on these findings can be found in Supplementary Information 11. Lastly, all isolates appeared to contain OmpA within their OMVs which is a multifunctional membrane protein and known modulator of infection and virulence determinants (Wang, 2002; Ortiz-Suarez et al., 2016).

### Use of FimA as a Potential Delivery Tag in *Escherichia coli* OMVs

A major motivation for understanding OMV synthesis in bacterial strains is to manipulate the cargo to enrich the OMVs for chosen proteins and other small molecules. Herein, we have shown that FimA and FliC appear to be selectively enriched in the OMVs depending on the prevailing regulatory conditions. We speculated that it should be possible to selectively target proteins to the OMVs using FimA although we were not clear whether FimA alone was sufficient or what part of the protein was necessary to facilitate correct targeting. We obtained and created two proteins that were differentially tagged with either GFP (chromosomal FimA + GFP) or Neon Green (exogenously expressed FimA + Neon Green as outlined in section “Materials and Methods”). We found that use of the two constructs gave two contrasting results.

**FIGURE 6:**
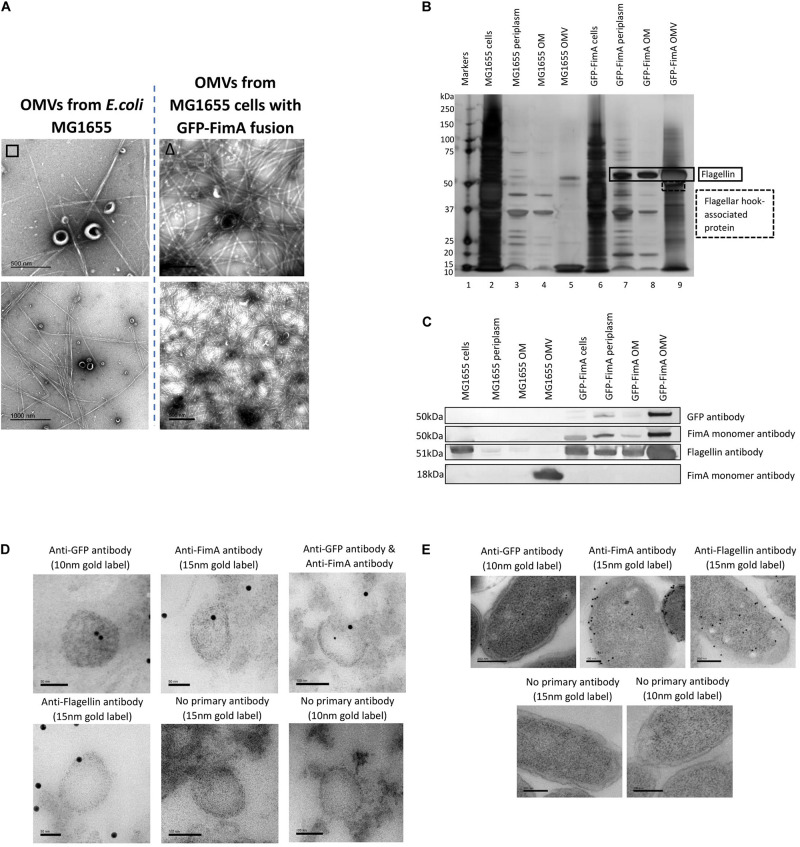
The transport and packaging of a GFP-FimA fusion protein in *E. coli* MG1655. **(A)** OMV co-purification with either fimbriae or flagella; **(B)** Proteome of the OMVs and **(C)** Western blot using anti FimA (monomer and polymerized) and anti-Flagellin antibodies. **(D,E)** TEM analysis of thin-sectioned OMVs **(D)** and *E. coli* FimA-GFP strain cells **(E)** embedded in resin. The sections were immunogold labeled and probed with: anti-GFP antibody, anti-FimA monomer antibody, anti-FimA monomer/anti-GFP antibodies mixed and anti-Flagellin antibody. As a negative control, the embedded OMVs were incubated in TBST only (no primary antibody). The samples were then incubated with the following secondary antibodies: 15 nm gold label or 10 nm gold label.

Firstly, Figure 6A indicates that fimbriae production in the *E. coli* MG1655 FimA-GFP (Adiciptaningrum et al., 2009) was disrupted and this led to the production of flagella, which was found co-purified with the OMVs. This was in contrast to the WT MG1655 strain which produced fimbriae only. When the purified OMVs were further analyzed (Figures 6B,C), it appeared that OMVs from the FimA-GFP fusion strain successfully contained the FimA-GFP fusion protein at 50 kDa. Interestingly, the OMVs also contained monomeric flagellin at 51 kDa and this appeared to be present at a higher concentration than the FimA-GFP fusion protein (Figures 6B,C and see Supplementary Information 5, Figure 6 for MS identification).

Using antibodies to both GFP and FimA, the FimA-GFP fusion protein was detected at approximately 50 kDa within the isolated OMV sample (Figure 6C) and at lower levels in the periplasm and OM. Therefore, the fusion of GFP to FimA chromosomally appeared to be sufficient for trafficking the FimA-GFP protein to the OMVs. Lastly, a transmission electron microscopy (TEM) analysis of thin-sectioned OMVs and cells embedded in resin was performed. The sections were immunogold labeled and probed with the following antibodies: (i) anti-GFP antibody, (ii) anti-FimA monomer antibody, (iii) both anti-FimA monomer and anti-GFP antibodies, and (iv) anti-Flagellin antibody (Figures 6D,E). These images support the conclusion that the FimA-GFP fusion protein and the Flagellin monomer protein were present within the OMVs from this strain.

**FIGURE 7:**
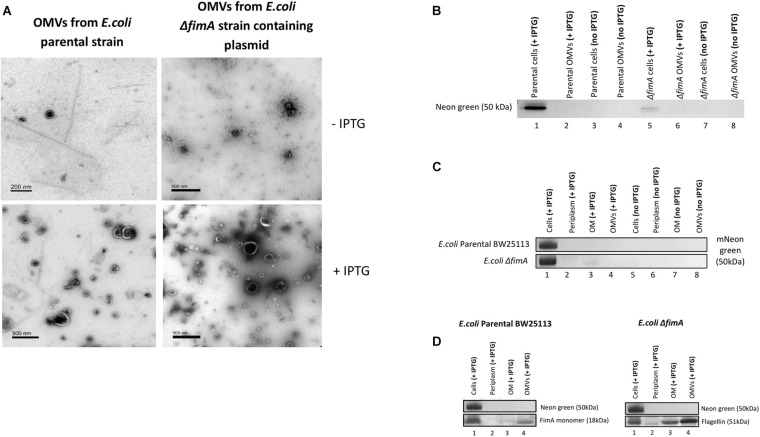
The transport and packaging of a mNeonGreen-FimA fusion protein in *E. coli* BW25113 and a Δ*fimA* mutant. **(A)** TEM images show purified OMVs from the *E. coli* BW25113 and Δ*fimA* mutant strains containing the pSB001 plasmid (encoding the mNeonGreen-FimA fusion protein) in presence or absence of IPTG. **(B)** Presence of mNeon green in either the cells or OMVs of the WT and Δ*fimA* mutant both in the presence or absence of IPTG induction. **(C)** Western blot using anti-Neon green on whole cells and subcellular [periplasm, outer membrane (OM) and OMV] fractions in the presence and absence of IPTG induction. **(D)** As C using anti-FimA monomer and anti-Flagellin antibodies.

A plasmid containing a FimA-mNeon green fusion protein (pSB001) was made and expressed in the *E. coli* (BW25133) and Δ*fimA* strain. When induced with IPTG, the cells of both strains appeared to hypervesiculate, giving rise to an increased yield of OMVs (Figure 7A). The proteome of the producer strains and the OMVs were analyzed by Western blotting. It was shown that the cells produced a FimA-Neon Green protein (when examined using an anti-Neon Green antibody) but they did not reach the OMVs (Figure 7B). This finding suggested that the Neon-Green-FimA construct was either incorrectly processed/targeted which was further supported by the absence of the construct in either the periplasm or the OM (Figures 7C,D).

Overall, the results using both the FimA-GFP and the FimA-NeonGreen constructs show that the disruption of the endogenous control circuits/regulation in some strains cause hypervesiculation and dysregulation in the processing and packaging of OMVs.

## Discussion

Unlike all previous studies into OMVs, we have focused the studies presented on the production of OMVs in *E. coli* WT strains that ordinarily produce either flagella (for motility) or fimbriae (for adherence and invasion) to give us an insight into the concurrent expression of OMVs and these important co-occurring cellular features. Previous studies have demonstrated that the production and regulation of these appendages are under complex control with flagella synthesis being dependent on a multitude of factors such as growth rate (Sim et al., 2017) and complex pulsatile regulatory networks that produce rich temporal dynamics and phenotypic heterogeneities (Kim et al., 2020). Similarly, it is recognized that Type 1 fimbrial production is a phase variable system that is produced uniformly throughout the growth curve but subject to regulatory controls such as CRP-cAMP (Müller et al., 2009). Taken as a whole, while *flhDC* and *lrhA* are recognized as central players in integrated transcriptional co-regulation of fimbriae and flagella (Lane et al., 2007), many complex regulatory circuits exist at both the cell and population level.

The assemblage of these extracellular structures (fimbriae and flagella) has been extensively reviewed elsewhere (Auvray et al., 2001; Nishiyama et al., 2005). The main structural subunits of these structures are formed by polymerization of monomers (FimA/FliC) that are delivered to the base of a growing filament in either the periplasm (fimbriae) or the cytoplasm (flagella). In this way, they are spatially separated and delivered *via* different mechanisms. Moreover, the function of fimbriae and flagella (principally invasion and motility) are mutually exclusive although there are suggestions that both are involved in adhesion and virulence events (Haiko and Westerlund-Wikström, 2013). Despite their ubiquity and co-occurrence in *E. coli* WT strains, it is noted that very few OMV studies acknowledge this and suggest that the purification of the OMVs by ultracentrifugation allows clean separation of the fimbriae or flagella.

A key finding in the present work was the clear demonstration of the presence of the major structural monomers [either FimA (from fimbriae) or FliC (from flagella)] in OMVs arising from *E. coli* K-12 strains. This is in contrast to *E. coli* strains that are stressed or engineered e.g., BL21 strains which are shown to hypervesiculate and appear to contain non-specific protein cargo (Figure 1). FimA and Flagellin are rarely found as monomers i.e., not in the polymerized form. FimA is the major structural protein in fimbriae and the monomers are synthesized and delivered to the bottom of the growing fimbriae by crossing the periplasm in a highly regulated chaperone/usher pathway in a wide variety of bacterial phyla (Liu and Ochman, 2007). FliC is the major filamentous protein in the flagella and is delivered to the growing filament within the MS ring/basal body present in the cytoplasmic membrane. Therefore, it was interesting to find monomeric FimA and Flagellin (that are formed in different regions of the cell) present in OMVs purified from WT strains.

It was also shown that the mutual exclusivity of the FimA (fimbrial monomer) and FliC (flagella monomer) is abrogated if some components of the regulation and assembly pathway were mutated/attenuated. As shown in Figures 2–5, the absence of components essential for the correct regulation and polymerization of FimA into fimbrial appendages (Δ*fim* B, C, D and F) predominantly prevented FimA packaging into OMVs. Additionally, if the “correct” WT packaging of FimA did not occur, then it was shown that FliC was packaged instead (Δ*fim* A, B, C, and F). When OMVs were analyzed from six clinical isolates, clinical isolate 5 OMVs contained FimA (but no Flagellin), and clinical isolate 6 contained Flagellin (but no FimA) which fits the mutual exclusivity hypothesis (Figure 5). However, OMVs from clinical isolate 3, Δ*fimE*, G, H, and I contained both FimA and FliC packaged within the OMVs so this is not a clear-cut story. See Supplementary Information 11 for further hypotheses on these findings.

Our findings led us to speculate on the possible advantages of the reciprocal regulation of fimbrial (FimA) and flagellar (FliC) monomers within *E. coli* OMVs. For example, it is likely to mirror the reciprocal regulation of adherence (by production of Type 1 fimbriae) and motility (by production of flagella) shown previously in UPEC *E. coli* (Cooper et al., 2012). In addition to this, it might further be reasoned on the basis of competing immunomodulation and immunevasion phenotypes for each of the two proteins in various hosts and niches:

FimA monomer – Immunoevasion/immunomodulation – as planktonic or adhering cells, it makes sense that an abundant protein (FimA) possesses gain of function to suppress host *via* OMV packaging. Its role has previously been demonstrated by Sukumaran et al. (2010) who suggested a FimA homolog could suppress host cell apoptosis by targeting a mitochondrial complex, the Bax-mediated release of cytochrome C. FimA has also been found in OMVs from other bacterial strains including *Porphyromonas gingivalis* (Mantri et al., 2015).

Flagellin monomer – Immunostimulation – Flagellin enrichment in OMVs has previously been shown in *E. coli* K12 (W3110) (Manabe et al., 2013), Enterotoxigenic *E. coli* (ETEC), and *Pseudomonas aeruginosa* PA01 and S470, a clinically relevant strain originating from a cystic fibrosis patient (Bauman and Kuehn, 2006). Flagellin is a virulence factor that is recognized by the innate immune system and known to induce a pro-inflammatory immune response in mice (Zgair, 2012), recognition by the NAIP5/NLRC4 inflammasome (Hajam et al., 2017) and known to bind both TLR-5, activating NF–κB signaling (Yoon et al., 2012) and TLR-11 (Hatai et al., 2016). Counter to this, there is clear evidence that, in *Salmonella*, while flagella interacts with TLR5, monomeric flagellin does not (Hayashi et al., 2001).

Finally, the present work sought to use the knowledge gained in this study to engineer a strain capable of selectively transporting a cargo protein to the OMV. Two approaches were used in the study, a chromosomally modified FimA-GFP obtained from AMOLF in the Netherlands (Adiciptaningrum et al., 2009) and a construct of pSB001 FimA-mNeon Green (entire). Using the chromosomally modified FimA-GFP in *E. coli* MG1655, it was shown that the FimA-GFP construct (50 kDa) was packaged into the OMVs but at low concentration, with flagellin being the major protein in this engineered system (Figures 6B,C). Using the FimA-mNeon Green constructs (expressed using a plasmid) it was shown that the fusion proteins did not traffic as expected and were not packaged into OMVs. It may therefore be concluded that it may be possible to use FimA to target cargo to OMVs but only when correctly or endogenously regulating and expressing the protein. This is simply because OMV targeting and increased yield, the major criteria for biotechnological production and downstream harvest and utility, would be better achieved by use of hypervesiculating strains (e.g., BL21 and derivatives) with appropriate OMV targeting proteins such as BL21 (DE3) Δ*ompA* (Fantappié et al., 2014). If exceptionally there was a desire to use a whole-cell OMV producing system capable of co-producing flagella or fimbriae it would be beneficial to both induce and overexpress the target and cargo to maximize the yield of downstream OMVs. Such a design brief was shown not to be possible using the FimA protein fusions (Figures 6, 7) and it is concluded that if the endogenous regulation is circumvented the correct trafficking and packaging of OMVs breaks down limiting its potential utility.

Overall, the work presented in the present paper shows that:

(i) Outer membrane vesicle yield and proteome is tightly regulated in K-12 WT *E. coli* strains. While it may be possible to simply engineer hypervesiculation, this is shown to be at the cost of selectivity of packaging.
(ii) Monomeric FimA is consistently and selectively enriched and packaged in the *E. coli* WT strains including MG1655 and BW25113.
(iii) FimA and FliC appear to be reciprocally regulated and generally mirror the regulation of adherence (by expression of Type 1 fimbriae) and motility (by expressing flagella) in cells.
(iv) FimA packaging into OMVs is dependent upon other components of the *fim* operon. Mutation of genes *fimA, B, C, E, F, G, H, I* caused the packaging of FliC into OMVs, either exclusively or in combination with FimA.
(v) In a study of six *E. coli* K-12 clinical isolates, two isolate OMVs contained monomeric FimA and two isolates contained Flagellin. Two of these isolates contained either FimA or Flagellin packaged in a mutually exclusive way.
(vi) Using a chromosomally modified FimA-GFP in *E. coli* MG1655, it was shown that the FimA-GFP construct (50 kDa) was successfully targeted to the OMVs.
(vii) Using the FimA-mNeon Green constructs (expressed using a plasmid) it was shown that the fusion proteins did not traffic as expected and were not packaged into OMVs.

In summary, the present work shows that two of the major externally transported and polymerizable proteins in *E. coli*, FimA (the major protein in Type 1 fimbriae), and FliC/Flagellin (the major structural protein in flagella), are reciprocally regulated and can switch the dominant protein packaged in the OMVs when normal regulatory circuits are abrogated. The exact mechanism and reasons for the packaging of such proteins have been speculated upon herein but are likely to be strain and host dependent and will require further study.

## Data Availability Statement

The original contributions presented in the study are included in the article/Supplementary Material, further inquiries can be directed to the corresponding author/s.

## Author Contributions

SAB conducted all the experiments and contributed to writing of the manuscript. MS contributed to experimental approach, review and revision of data, and writing of manuscript. GKR designed the experiments, supervised the work of SAB, and wrote the manuscript. All authors contributed to the article and approved the submitted version.

## Conflict of Interest

The authors declare that the research was conducted in the absence of any commercial or financial relationships that could be construed as a potential conflict of interest.

## Footnotes

1 https://ecocyc.org/

2 https://www.genewiz.com/en-GB/
