# Supplementary material for: Reciprocal Packaging of the Main Structural Proteins of Type 1 Fimbriae and Flagella in the Outer Membrane Vesicles of “Wild Type” Escherichia coli Strains
Source: Front Microbiol. 2021 Feb 12;12:557455. doi: 10.3389/fmicb.2021.557455 (PMC7907004; doi:10.3389/fmicb.2021.557455)
Supplement: Supplementary file 1 [file Data_Sheet_1.docx]

**Supplementary Information**

**S1 – Further information on the main *E. coli* strains used in the study**

* In this paper, Type 1 pilus / Type 1 pili and Type 1 fimbriae are used interchangeably. For clarity, all quotes were changed to ‘Type 1 fimbriae’ or ‘T1F’

**S2 – Materials and Methods for TEM**

1. Embedding bacterial cells in resin for immunogold labelling and TEM analysis

1 ml overnight culture was harvested by centrifugation at 13,000 RPM for 2 minutes. All centrifugation steps were carried out at 13,000 RPM for 2 minutes at room temperature unless otherwise specified. Cells were fixed in 1.5 ml 2.5% glutaraldehyde in 100 mM sodium cacodylate buffer pH 7.2 (CAB) for 3 hours with gentle rotation. Fixed cells were pelleted then washed twice in 100 mM CAB buffer, followed by final resuspension in 1.5 ml CAB. Cells were pelleted then resuspended in 1.5 ml 50% ethanol then were mixed with gentle for 10 minutes. This was repeated so that the cells were washed in 70% ethanol, 90% ethanol and 3x washes in 100% ethanol to remove all water. The cell pellets were finally resuspended in 1.5 ml LR White Resin Medium Grade (Agar scientific) and left spinning on the rotor overnight at room temperature. Cells were pelleted then resuspended in fresh LR White Resin (Agar Scientific) twice. Cells resuspended in resin were left rotating at room temperature for 5 hours. Cells were pelleted and resuspended in 0.5 ml resin which were then added to Gelatin capsules (Agar Scientific). Fresh resin was used to top up the capsules to the point of overflowing. The capsules were centrifuged at 1000 RPM for 5 minutes then incubated at 60°C for 22 hours to allow polymerisation of resin inside the gelatin capsules. The gelatin capsules were removed and stored at room temperature.

(b) Embedding OMVs in resin for immunogold labelling and TEM analysis

*E. coli* strains were grown in 750 ml LB and purified using the standard OMV purification protocol*.* The final OMV pellet was resuspended in 1 ml HEPES buffer. OMVs were concentrated by centrifugation at 13,200 RPM for 30 minutes at 4°C (the standard for centrifugation in this protocol). OMVs were fixed in 1.5 ml 2.5% glutaraldehyde in 100 mM sodium cacodylate buffer pH 7.2 (CAB) for 1.5 hours with gentle rotation. OMVs were pelleted then washed twice in 100 mM CAB, followed by a final resuspension in 25 μl CAB. The OMVs in CAB were incubated at 54**°**C in a water bath. After warming, 25 μl of pre-heated 3% agarose solution in 100 mM CAB was added to the OMV suspension and mixed thoroughly. The agarose-OMV suspension was then transferred into a pre-warmed frame constructed from two glass microscope slides separated by an acetate gasket. The slides were incubated at 4°C for 10 minutes to set. The microscope slides were separated and the agarose gel was cut into 2mm x 2mm squares. The agarose pieces were treated with 0.1% Alcian blue in 1% acetic acid then washed twice in 3 ml 100 mM CAB. Using this process, agarose pieces were washed in 50% ethanol, 70% ethanol, 90% ethanol and finally 3x in 100% ethanol. All 100% ethanol was removed and agarose pieces were left in 3 ml resin overnight at room temperature. All resin was removed around the agarose pieces then fresh resin was added. This was repeated and the agarose pieces were left in resin for 5 hours. This was then added to Gelatin capsules which were filled with resin to the top until overflowing. 1 agarose piece (dyed blue) was added to the bottom of the gelatin capsule filled with resin. Capsules were incubated at 60°C for 22 hours to allow polymerisation. The gelatin capsules were removed and stored at room temperature.

**(c) Sectioning and visualisation of embedded samples.**

After embedding, samples were ultra-thin sectioned on a RMC MT-XL ultra-microtome with a diamond knife (diatome 45°). Sections were placed on un-coated gold grids (300 mesh size). Grids were washed in 2% BSA in TBST for 1 minute then 30 minutes. Grids were incubated in 15 μl primary antibody (diluted 1:50 in TBST) at 4°C overnight. Grids were subject to 5 x 1 minute washes in TBST then incubated in the relevant secondary immunogold-conjugated antibody (diluted 1:50 in TBST) for 30 minutes. Grids were then washed 5 x 1 minute in TBST then 5 x 1 minute in water. The grids were incubated in 4.5% uranyl acetate in 1% acetic acid solution for 15 minutes then washed in a stream of ultrapure water. Lastly, grids were stained by incubation in Reynolds lead citrate for 3 minutes then washed in a stream of ultrapure water. Grids were air dried for 30 minutes then loaded on to the TEM for analysis.

**S3 - Dilutions for primary and secondary antibodies during Western blotting and Immunogold labelling TEM**

# S4 - Mass spectrometry (matrix-assisted laser desorption/ionisation, MALDI)

SDS-PAGE gels containing the bands of interest were subject to 2 x 10 minute washes with ultrapure water. Bands of interest were then carefully excised from the SDS-PAGE with a clean washed scalpel and cut further into 1mm x 1mm squares. A protocol for in-gel digestion was carried out as described in Shevchenko *et al.* 2006. Proteins were identified using Bruker ultrafleXtreme MALDI-TOF/TOF mass spectrometer and associated software. Samples to be identified were added to the MTP Anchorchip MALDI-TOF plate (Bruker). 0.5 μl sample was added to the MALDI-TOF plates in known coordinates and left to air dry. 1 μl matrix solution (0.7 mg/ml α-Cyano-4-hydroxycinnamic acid dissolved in solvent mixture 85% acetonitrile, 15% water, 0.1% TFA and 1 mM NH_4_H_2_PO_4_) was then added on top of each sample and left to air dry. 0.5 μl Peptide Calibration Standard (Bruker, catalog # 8222570) was added to the plate and left to air dry. The plate was loaded on to the instrument and the following settings were used: **Polarity:** positive, **Laser frequency:** 2 kHz, **Ion sources:** 25 kV and 22.35 kV, **Lens:** 7.5 kV, **Pulsed ion extraction:** 80 nS, **Peptide Calibration** **Range:** 700-3500 Da, **Data sampling rate:** 4 Gs/s. For each sample 3500 shots were summed and saved. Protein was identified by a Peptide Mass Fingerprint (PMF) search in the Mascot database. A match is significant if it has a score greater than 70.

**S5 - Plasmid (pSB001) details**

The plasmid pJB005 was donated by Dr Alex Moores (UKC) for use in cloning. See below for plasmid map and details. The vector is a derivative of the pCA24N plasmid (Kitagawa *et al*. 2005) with mNeonGreen fused at the C-terminal, inserted between cut sites NdeI and AscI and fused with mNeon Green protein with 6x His-tag. The plasmid was re-named pSB001.


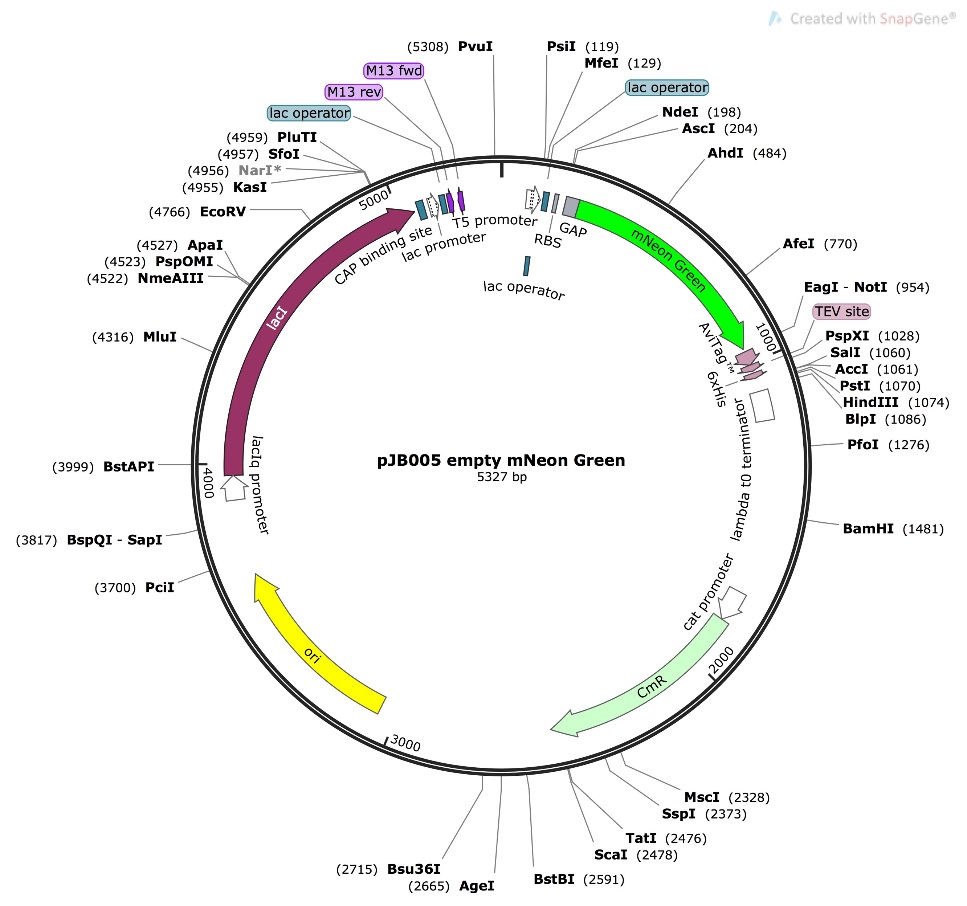


Table 1 - List of primers ordered from IDT with brief descriptions for their use

**S6 - Methods used for identification of fimbriae and/or flagella co-purified with the OMVs**

**Using TEM to identify fimbriae and flagella**

TEM images were compared of purified OMVs from *E. coli* strains known to produce flagella (*ΔfimA* and FimB-LacZ) and strains known to produce fimbriae (BW25113, *ΔfliC,* MG1655 and fimbriae locked on). TEM alone was used to detect flagella in OMV samples as they are very distinctive. Figure 1 below shows that flagella are thicker and longer than fimbriae and are also curvy/wavy. Type 1 fimbriae were found to be short, thin and straight appendages (compare S6, Figure 1 **a** and **d** with **b, c, e, f**) when compared with flagella.


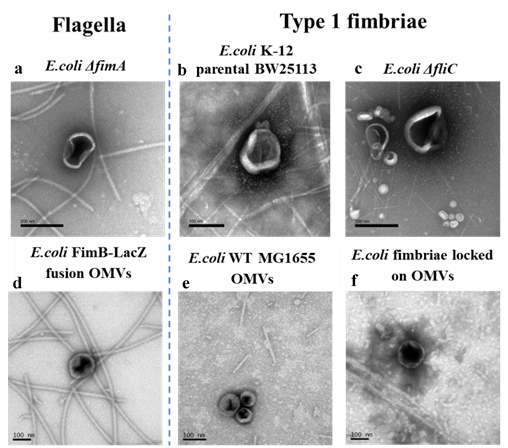


S6, Figure 1 TEM images of OMVs purified from six *E. coli* strains to compare co-purification of flagella and fimbriae

TEM analysis of purified OMVs from *E. coli* strains: **a** *ΔfimA,* **b** Parental BW25113, **c** *ΔfliC,* **d** FimB-LacZ fusion, **e** MG1655 and **f** fimbriae production locked on strain.

**Western blotting with anti-polymerised FimA to detect fimbriae**


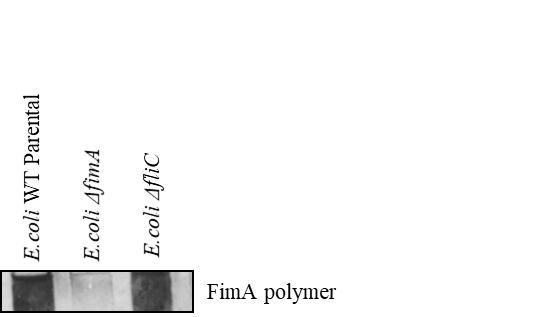
FimA monomers polymerise to form the main structural subunit of fimbriae. These polymers are so stable that they do not depolymerise during SDS-PAGE and are therefore too large to migrate through the gel. Polymerised FimA/Type 1 fimbriae were detected in the wells of Western blots when probing with the anti-FimA polymer antibody. OMVs purified from *ΔfimA* were used as a negative control and *ΔfliC* OMVs were used as a positive control (S6, Figure 2).

S6, Figure 2 Polymerised FimA positive and negative Western blot controls

A Bradford assay was performed and all samples were standardised to be the same protein concentration. TCA precipitation was used to concentrate samples prior to loading on an SDS-PAGE gel. Purified OMV samples were probed using anti-FimA polymer antibody. Image was cropped to show positive and negative controls for this antibody.

Fimbriae agglutination test with *E. coli* cells to detect fimbriae

Another method used to determine whether *E. coli* cells (from which the OMVs are purified from) express functional and intact fimbriae was a simple yeast agglutination test. The *E. coli* cells of interest were mixed 1:1 with *Saccharomyces cerevisiae* cells on a glass microscope slide. If fimbriae are present, FimH (the adhesin on the tip of Type 1 fimbriae) adheres to the *S. cerevisiae* cells. This causes the agglutination of the yeast cells which was visualised by light microscopy (S6, Figure 3 **a** and **b)**.

S6, Figure 3 Yeast agglutination test to detect fimbriae expression on *E. coli* cells

*Saccharomyces cerevisiae* were mixed 1:1 with the following *E. coli* strains: fimbriae locked on (**a**), MG1655 (**b**), FimB-LacZ fusion (**c**), BL21 (**d)**, BL21 (DE3) (**e**), LB only (**f**). Yeast cell agglutination was visualised using light microscopy at 400x magnification. Green ticks indicate yeast cell agglutination and therefore fimbriae.


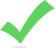

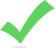


**c**


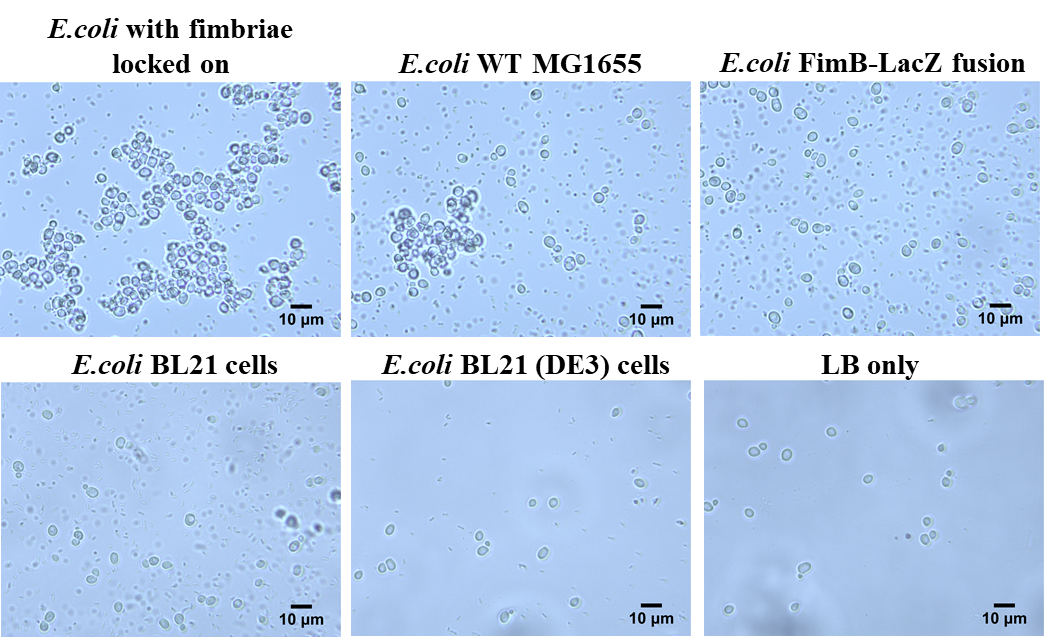


**a**

**b**

**e**

**d**

**f**

**S6, Table 1 - Summary table to confirm which strains produce fimbriae and/or flagella using all available strain evidence.**

Purple indicates which strains express fimbriae only, yellow indicates which strains produce flagella only and orange represents strains that produce both fimbriae and flagella together.

S7 - Identification of proteins of interest by mass spectrometry

Bands of interest were excised from silver stained SDS-PAGE gels then identified by mass spectrometry. Proteins were identified by a Peptide Mass Fingerprint (PMF) search in the Mascot database. A match is significant if it has a score greater than 70.

**Figure 1**

| **Lane number** | **Strain protein originated from** | **Protein detected (using MS/MS)** | **Score (score needed to be significant)** | **Protein MW (kDa)** | **Protein reference code** |
| --- | --- | --- | --- | --- | --- |
| 3 | *E. coli* BL21 (DE3) | Outer membrane protein F OS=Escherichia coli (strain K12) | 125 (70) | 39.309 | OMPF_ECOLI |
| 8 | *E. coli* WT MG1655 | Type-1 fimbrial protein, A chain OS=Escherichia coli (strain K12) | 71 (70) | 18.214 | FIMA1_ECOLI |
| 10 | *E. coli* FimB-LacZ fusion | Flagellin OS=Escherichia coli (strain K12) | 255 (70) | 51.265 | FLIC_ECOLI |

**Figure 2B**

| **Lane number** | **Strain protein originated from** | **Protein detected (using MS/MS)** | **Score (score needed to be significant)** | **Protein MW (kDa)** | **Protein reference code** |
| --- | --- | --- | --- | --- | --- |
| 3 | *E.coli ΔfimA* | Major outer membrane prolipoprotein Lpp OS=Escherichia coli (strain K12) | 108 (39) | 8 | LPP_ECOLI |
| 4 | *E.coli ΔfimA* | Flagellin OS=Escherichia coli (strain K12) | 341 (39) | 51 | FLIC_ECOLI |
| 6 | *E.coli ΔfliC* | Major outer membrane prolipoprotein Lpp OS=Escherichia coli (strain K12) | 128 (39) | 8 | LPP_ECOLI |
| 7 | *E.coli ΔfliC* | Type-1 fimbrial protein, A chain OS=Escherichia coli (strain K12) | 79 (39) | 18 | FIMA1_ECOLI |
| 7 | *E.coli ΔfliC* | Antigen 43 OS=Escherichia coli (strain K12) | 71(39) | 107 | AG43_ECOLI |

**Figure 3**

| **Lane number** | **Strain protein originated from** | **Protein detected (using MS/MS)** | **Score (score needed to be significant)** | **Protein MW (kDa)** | **Protein MW (kDa)** |
| --- | --- | --- | --- | --- | --- |
| 3 | *E.coli* parental BW25113 | Antigen 43 | 148 (70) | 106818 | AG43_ECOLI |
| 3 | *E.coli* parental BW25113 | FimA | 71 (70) | 18214 | FIMA1_ECOLI |
| 4 | *E.coli* *ΔfimA* | Flagellin | 214 (70) | 51265 | FLIC_ECOLI |
| 7 | *E.coli* *ΔfimE* | FimA | 61 (41) | 18214 | FIMA1_ECOLI |
| 8 | *E.coli ΔfimF* | Flagellin | 267 (70) | 51265 | FLIC_ECOLI |
| 10 | *E.coli ΔfimH* | FimA | 55 (41) | 18214 | FIMA1_ECOLI |
| 4 | *E.coli ΔfimA* | Flagellin | 288 (70) | 51265 | FLIC_ECOLI |
| 6 | *E.coli ΔfimI* | Flagellin | 256 (70) | 51265 | FLIC_ECOLI |
| 6 | *E.coli ΔfimI* | FimA | 59 (40) | 18214 | FIMA1_ECOLI |
| 8 | *E.coli* WT MG1655 | Antigen 43 | 120 (70) | 106818 | AG43_ECOLI |
| 8 | *E.coli* WT MG1655 | FimA | 57 (41) | 18214 | FIMA1_ECOLI |
| 9 | *E.coli* WT MG1655 FimB-LacZ fusion | Flagellin | 236 (70) | 51265 | FLIC_ECOLI |
| 10 | *E.coli* WT fimbriae locked on | Flagellin | 257 (70) | 51265 | FLIC_ECOLI |
| 10 | *E.coli* WT fimbriae locked on | FimA | 71 (70) | 18214 | FIMA1_ECOLI |

**Figure 4**

| **Lane number** | **Strain protein originated from** | **Protein detected (using MS/MS)** | **Score (score needed to be significant)** | **Protein MW (kDa)** | **Protein reference code** |
| --- | --- | --- | --- | --- | --- |
| 2 | *E.coli* WT parental BW25113 | Antigen 43 OS=Escherichia coli (strain K12) | 522 (40) | 106.818 | AG43_ECOLI |
| 2 | *E.coli* WT parental BW25113 | Type-1 fimbrial protein, A chain OS=Escherichia coli (strain K12) | 480 (39) | 18.214 | FIMA1_ECOLI |
| 3 | *E.coli* *ΔfimA* | Flagellin OS=Escherichia coli (strain K12) | 214 (70) | 51265 | FLIC_ECOLI |
| 5 | *E.coli* *ΔlrhA* | Flagellin OS=Escherichia coli (strain K12) | 1036 (40) | 51.265 | FLIC_ECOLI |
| 5 | *E.coli* *ΔlrhA* | Type-1 fimbrial protein, A chain OS=Escherichia coli (strain K12) | 244 (39) | 18.214 | FIMA1_ECOLI |

**Figure 5**

| Lane number | Strain protein originated from | Protein detected (using MS/MS) | Score (score needed to be significant) | Protein MW (kDa) | Protein reference code |
| --- | --- | --- | --- | --- | --- |
| 2 | *E.coli* WT parental BW25113 | Antigen 43 OS=Escherichia coli (strain K12) | 522 (40) | 106.818 | AG43_ECOLI |
| 2 | *E.coli* WT parental BW25113 | Type-1 fimbrial protein, A chain OS=Escherichia coli (strain K12) | 480 (39) | 18.214 | FIMA1_ECOLI |
| 3 | *E.coli ΔfimA* | Flagellin OS=Escherichia coli (strain K12) | 486 (39) | 51.265 | FLIC_ECOLI |
| 4 | *E.coli ΔfliC* | Type-1 fimbrial protein, A chain OS=Escherichia coli (strain K12) | 454 (39) | 18.214 | FIMA1_ECOLI |
| 6 | Clinical isolate 2 | Outer membrane protein A OS=Escherichia coli (strain K12) | 93 (40) | 37.292 | OMPA_ECOLI |
| 7 | Clinical isolate 3 | Flagellin OS=Escherichia coli (strain K12) | 84 (70) | 51.265 | FLIC_ECOLI |
| 7 | Clinical isolate 3 | F7-2 fimbrial protein OS=Escherichia coli O6:H1 | 53 (39) | 19.287 | FMF2_ECOL6 |
| 8 | Clinical isolate 4 | KS71A fimbrillin OS=Escherichia coli | 66 (38) | 19.412 | FMK1_ECOLX |
| 9 | Clinical isolate 5 | Type 1 fimbrin D-mannose specific adhesin OS=Escherichia coli (strain K12) | 182 (39) | 31.682 | FIMH_ECOLI |
| 9 | Clinical isolate 5 | Type-1 fimbrial protein, A chain OS=Escherichia coli (strain K12) | 379 (39) | 18.214 | FIMA1_ECOLI |
| 10 | Clinical isolate 6 | Flagellin OS=Escherichia coli (strain K12) | 212 (40) | 51.265 | FLIC_ECOLI |

**Figure 6**

| Lane number | Strain protein originated from | Protein detected (using MS/MS) | Score (score needed to be significant) | Protein MW (kDa) | Protein reference code |
| --- | --- | --- | --- | --- | --- |
| 9 | *E.coli* GFP-FimA fusion | Flagellin OS=Escherichia coli (strain K12) | 1147 (40) | 51.265 | FLIC_ECOLI |
| 9 | *E.coli* GFP-FimA fusion | Flagellar hook-associated protein 2 OS=Escherichia coli (strain K12) | 194 (40) | 48.427 | FLID_ECOLI |

**S8 - Proteinase K test to evidence that Flagellin is protected within OMVs**


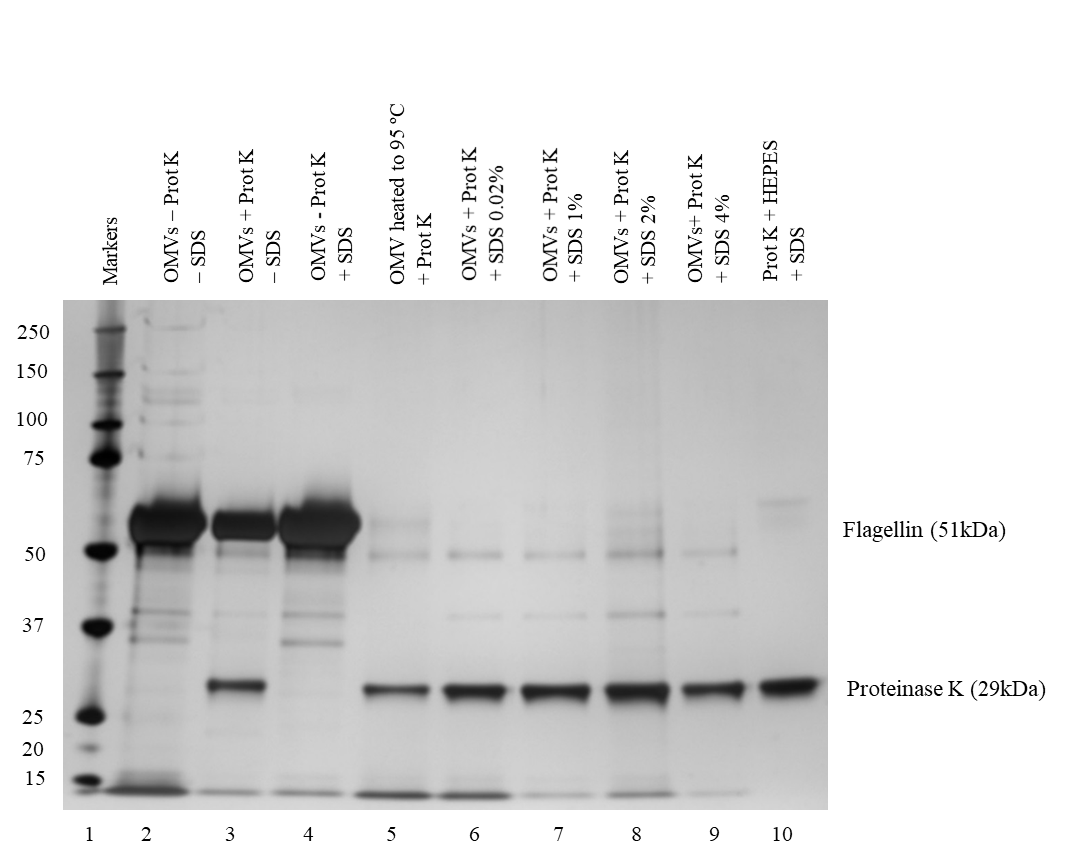


Flagellin (51 kDa)

Proteinase K (29 kDa)

kDa

Figure S8 Proteinase K test on OMVs from *E. coli* FimB-LacZ fusion strain

OMVs were incubated in the presence and absence of 10 µg/ml Proteinase K and various concentrations of SDS for 30 mins at 37ºC. 5 mM PMSF was added to inhibit Proteinase K and samples were incubated for another 30 mins at 37ºC. TCA precipitation was used to concentrate samples prior to loading on an SDS-PAGE gel. The SDS-PAGE gel was run then silver stained to visualise the OMV protein profile.

**S9 - Final summary tables to compare mutual exclusivity of FimA and Flagellin packaging in OMVs from various *E. coli* strains (K-12 and B strains)**. Table **a** is a summary of the findings from all strains tested. Table **b** summarises the percentage of *E. coli* strains that give rise to OMVs with FimA and Flagellin packaged in a mutually exclusive way. Table gives **c** the percentage of *E. coli* strains that give rise to OMVs with FimA and Flagellin packaged in a mutually exclusive way **excluding** from the data set any strains do not contain FimA or Flagellin in their OMVs.

**a**

**b**

**c**

**S10 - Summary table to compare the packaging of FimA and Flagellin monomers into OMVs** with the production of fimbriae and/or flagella in the parental strain. The criteria for a ‘match’ is explained in Table **a** and the results are found in **b**. Type 1 fimbriae is abbreviated to ‘T1F’.

**a**

**b**

There are 7 instances out of 27 strains in total (26%) where packaging of FimA and/or Flagellin monomers into OMVs is independent of whether fimbriae or flagella are expressed on the parent cell.

**S11 – Comparison of OMVs purified from a range of *E. coli* strains with deletions of various fimbriae or flagella associated proteins and discussion of results.** In the following tables, Type 1 fimbriae is abbreviated to ‘T1F’.

**a**

**b**

**c**

**Additional references for Supplementary Information**

Bange, G., Kümmerer, N., Engel, C., Bozkurt, G., Wild, K. and Sinning, I. (2010). FlhA provides the adaptor for coordinated delivery of late flagella building blocks to the type III secretion system. *Proc Natl Acad Sci U S A.,* 107(25), 11295–11300. doi: 10.1073/pnas.1001383107.

Galeva, A., Moroz, N., Yoon, Y.H., Hughes, K.T., Samatey, F.A. and Kostyukova, A.S. (2014). Bacterial Flagellin-specific chaperone FliS interacts with anti-sigma factor FlgM. *Journal of Bacteriology,* 196(6), 1215-1221. doi: 10.1128/JB.01278-13.

Kitagawa, M., Takeshi, A., Arifuzzaman, M., Ioka-Nakamichi, T., Inamoto, E., Toyonagwa, H. and Mori, H. (2005). Complete set of ORF clones of *Escherichia coli* ASKA library (A Complete Set of *E. coli* K -12 ORF Archive): Unique Resources for Biological Research. *DNA Res.* 12, 291–299. doi: 10.1093/dnares/dsi012

Postel, S., Deredge, D., Bonsor, D.A., Yu, X., Diederichs, K., Helmsing, S., Vromen, A., Friedler, A., Hust, M. Egelman, E.H., Beckett, D., Wintrode, P.L. and Sundberg, E.J.(2016). Bacterial flagellar capping proteins adopt diverse oligomeric states. *eLife,* 5:e18857. doi: 10.7554/eLife.18857.

Ribeiro, C.A., Rahman, L. A., Holmes, L. G., Woody, A.M., Webster, C. M., Monaghan, T.I., Robinson, G.K., Muhlschlegel, F.A.M., Goodhead, I.B. and Shepherd, M. (2021) Nitric oxide (NO) elicits aminoglycoside tolerance in *Escherichia coli* but antibiotic resistance gene carriage and NO sensitivity have not co-evolved. *Archives in Microbiology*. In press.

Schwan, W.R. (2011). Regulation of *fim* genes in uropathogenic *Escherichia coli. World J Clin Infect Dis,* 1(1), 17–25. doi: 10.5495/wjcid.v1.i1.17.

Svensson, S.L., Pryjma, M. and Gaynor, E.C. Flagella-Mediated Adhesion and Extracellular DNA Release Contribute to Biofilm Formation and Stress Tolerance of *Campylobacter jejuni.* (2014) *PLoS One* 9(8), e106063. doi: 10.1371/journal.pone.0106063.
